# Modified gemcitabine plus nab‐paclitaxel regimen in advanced pancreatic ductal adenocarcinoma

**DOI:** 10.1002/cam4.3229

**Published:** 2020-06-09

**Authors:** Jane E. Rogers, Jonathan D. Mizrahi, Lianchun Xiao, Chirayu Mohindroo, Rachna T. Shroff, Robert Wolff, Gauri R. Varadhachary, Milind M. Javle, Michael Overman, David R. Fogelman, Kanwal P. S. Raghav, Shubham Pant, Florencia McAllister

**Affiliations:** ^1^ Pharmacy Clinical Programs University of Texas MD Anderson Cancer Center Houston TX USA; ^2^ Department of Cancer Medicine University of Texas MD Anderson Cancer Center Houston TX USA; ^3^ Department of Biostatistics University of Texas MD Anderson Cancer Center Houston TX USA; ^4^ Department of Clinical Cancer Prevention University of Texas MD Anderson Cancer Center Houston TX USA; ^5^ Department of Gastrointestinal Medical Oncology University of Texas MD Anderson Cancer Center Houston TX USA; ^6^ Department of Investigation Cancer Therapeutics University of Texas MD Anderson Cancer Center Houston TX USA; ^7^ Clinical Cancer Genetics Program University of Texas MD Anderson Cancer Center Houston TX USA; ^8^ Present address: University of Arizona Cancer Center Phoenix AZ USA

**Keywords:** carcinoma, pancreatic ductal, gemcitabine, nab‐paclitaxel, pancreatic neoplasms

## Abstract

**Background:**

Gemcitabine (GEM) plus nab‐paclitaxel (NabP) (GEM 1000 mg/m^2^ IV over 30 minutes + NabP 125 mg/m^2^ IV given days 1, 8, and 15 every 28 days) is one of the two standard of care combination therapies for metastatic pancreatic ductal adenocarcinoma (PDAC). Our cancer center has utilized GEM‐NabP given every two‐weeks due to tolerability and patient convenience. Here, we review the safety and efficacy of this modified regimen.

**Methods:**

Metastatic PDAC patients (pts) who initiated front‐line or second‐line GEM‐NabP during 2013‐2017 were retrospectively reviewed. Primary objective was overall survival. Secondary objectives were disease control rate, progression‐free survival, and the incidence of dose delays and/or adjustments.

**Results:**

From a total of 235 patients, 140 pts received GEM‐NabP front‐line while 95 pts received GEM‐NabP second‐line. Median dosing was 600 mg/m^2^ at fixed‐dose rate for GEM and 125 mg/m^2^ for NabP given predominantly (~90%) every two‐weeks. Eastern Cooperative Group performance status of 0 and 1 pts had front‐line OS of 12.7 and 9.6 months and when given second‐line had OS of 8 months and 7.3 months, respectively. ECOG 0 and 1 pts had front‐line progression‐free survival (PFS) of 5.3 months and 2.8 months and second‐line PFS was 3.5 months and 2.4 months, respectively. Treatment was well tolerated with limited dose modifications.

**Conclusion:**

Our analysis revealed safety with every two‐week low dose GEM‐NabP while maintaining efficacy. Patient schedule convenience should factor into metastatic incurable malignancies. We suggest the use of every two‐week GEM‐NabP particularly in patients desiring a modified schedule.

## Graphical abstract

Our real‐world data, outside the context of a clinical trial, suggests that every two‐week Gem‐NabP can be administered safely without compromising efficacy in patients with metastatic pancreatic adenocarcinoma (PDAC). In particular, the alternate (bi‐weekly) dosing regimen showed low rates of grades 3 and 4 toxicities and no treatment‐related deaths. This regimen is better tolerated than weekly Gem‐NabP, is convenient, and allows similar palliative benefits to patients with metastatic PDAC.

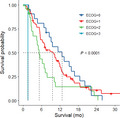

## 1 INTRODUCTION

Pancreatic ductal adenocarcinoma (PDAC) accounts for 90% of pancreatic cancers.^1^, ^2^, ^3^ PDAC continues to carry a dismal prognosis and represents the fourth most common cause of cancer‐related deaths in both the United States and Europe.^1^, ^4^ Overall 5‐year survival rate is 9% and declines further to less than 3% in patients with distant disease.^5^ In the Unites States, pancreatic cancer is projected to be the second most common cause of cancer related death by 2030.^3^

Historically, gemcitabine (GEM) monotherapy was the standard front‐line therapy approved based on clinical benefit and limited survival improvement for metastatic patients.^6^ GEM‐based chemotherapy combinations trials were conducted following GEM approval; however, a standard combination failed for years to emerge, and treatment advancement stalled until 2011. At that time, Conroy et al established a pivotal metastatic PDAC management advancement.^7^ The investigators conducted a phase II‐III multicenter, randomized controlled trial in patients with an Eastern Cooperative Oncology Group performance status of 0 or 1 comparing standard of care GEM alone to a fluoropyrimidine combination regimen of 5‐fluorouracil + leucovorin +oxaliplatin + irinotecan (FOLFIRINOX). Median OS and progression‐free survival (PFS) were improved in the FOLFIRINOX arm (*P* < .001). Median OS advantage was approximately 4 months (FOLFIRINOX median OS 11.1 months vs GEM median OS 6.8 months, *P* < .001) and median PFS advantage of 3 months (FOLFIRINOX median PFS 6.4 months vs GEM median PFS 3.3 months, *P* < .001). Front‐line FOLFIRINOX became the standard of care for metastatic PDAC patients able to tolerate intensive therapy. In 2013, Von Hoff et al (MPACT trial) reported the results of a multicenter phase III‐randomized trial in patients with Karnofsky performance status score of 70 or more comparing GEM + nab‐paclitaxel (NabP) to GEM alone in metastatic PDAC.^8^ The combination consisted GEM 1000 mg/m^2^ intravenous (IV) over 30 minutes + NabP at 125 mg/m^2^ IV given over 30 minutes on days 1, 8, and 15 every 4 weeks. Similar to FOLFIRINOX, the combination regimen improved outcomes compared to GEM alone. Median OS advantage was ~2 months (Gem‐NabP median OS 8.5 months vs GEM median OS 6.7 months, *P* < .001) and median PFS advantage was ~2 months (Gem‐NabP median PFS 5.5 months vs GEM median PFS 3.7 months, *P* < .001). Thus, Gem‐NabP became a second standard of care option for metastatic PDAC. The combination did result in the need for dose reductions in NabP for 41% of patients, mostly due to neurotoxicity, and in GEM for 47% of patients, mostly due to neutropenia and leukopenia. This regimen allowed for an alternate front‐line combination to FOLFIRINOX.

Both combination regimens (FOLFIRINOX and GEM + NabP) are incorporated currently in the front‐line treatment of locally advanced and metastatic PDAC.^1^, ^2^ At our institution, GEM‐NabP is often prescribed in an every two‐week schedule for tolerability and patient convenience allowing for decreased infusion times and follow‐up visits. The purpose of this retrospective analysis was to review the safety and efficacy of this every two‐week regimen while characterizing our dosing practices in metastatic PDAC patients.

## 2 MATERIALS AND METHODS

Our study was a single institution, retrospective chart review of patients with metastatic PDAC who received GEM‐NabP front‐line or second‐line. Adult metastatic PDAC patients who initiated this regimen from June 1, 2013 to July 1, 2017 were included. Patients must have received this treatment at our center along with radiographic follow‐up every 8‐12 weeks at our center. Patients who received recommendations from our center but received therapy elsewhere were excluded. Patients with unresectable locally advanced disease were excluded. OS was our primary objective. Secondary objectives were PFS and disease stability/regression (disease control) vs progression on first radiographic evaluation. Any response or stable disease by radiology review was classified as disease control. Toxicity was evaluated based on the need for dose delays/reductions or when patients were admitted. The reasons for dose delays and admissions were collected. Common Terminology Criteria for Adverse Effects (CTCAE) version 4^9^ was utilized to determine adverse effect grade retrospectively when toxicities requiring dose delays/reductions or requiring admission occurred.

Data collection included patient demographics (age, gender, race, ECOG performance status) and tumor characteristics (metastatic disease sites, primary tumor location). Treatment factors collected were GEM‐NabP starting date, starting dose, and chemotherapy schedule. Patients who initiated a 3 week on 1 week off schedule were reviewed whether the schedule was modified to an every two‐week schedule. Patients who received GEM‐NabP second‐line had their first‐line regimen documented and whether they received therapy in the third‐line setting. Additionally, date of progression, date of death or last follow‐up, and the incidence and reason for dose delays and/or adjustments were included.

### 2.1 Ethics

Our institutional review board approved our study. A waiver of consent was granted given the minimal risk of a retrospective evaluation.

### 2.2 Statistical analysis

Statistical analysis was performed using descriptive statistics. Continuous variables were described using median and range while categorical data were summarized using frequencies and percentages. Chi‐squared test/ Fisher's exact test and Wilcoxon rank sum test were used to evaluate the association between response and patient characteristics. The Kaplan‐Meier method was used to estimate survival outcomes, the log rank test and univariable Cox models were applied to evaluate the association between survival outcomes and covariates. OS, the primary objective, was calculated as the time between treatment start to death or last follow‐up date. PFS was defined as the time between treatment start date to treatment discontinuation for progressive disease, performance status decline, toxicity, patient preference, or death/last follow‐up. Disease control was defined as any response plus stable disease at first radiographic scan.

## 3 RESULTS

### 3.1 First‐line GEM‐NabP results

One hundred and forty patients received GEM‐NabP in the front‐line setting. Median age was 67 yo with just over half (58.6%) male. Most patients (71.4%) were Caucasian and had an ECOG performance status of 0‐1 (65%). Patient characteristics and demographics are listed in Table 1. Primary tumor location most often was in the head of the pancreas, and half of patients had metastatic disease to more than one organ. Most common organs involved were liver, peritoneum, and lung. Other sites include adenopathies, bone, ascites, adrenal, pleura, ovaries, breast, and spleen. Median dosing of GEM was 600 mg/m^2^ (range: 400 mg/m^2^ to 750 mg/m^2^) given over 10 mg/m^2^/minute + NabP at 125 mg/m^2^ (range: 65 mg/m^2^ to 200 mg/m^2^) given over 30 minutes. Of note, our institution's standard of care practice is to administer GEM at a fixed‐dose rate (10 mg/m^2^/min) to maximize GEM phosphorylation as described in the E6201 trial^10^ while our institution additionally reduces the GEM dose to 600 mg/m^2^ or 750 mg/m^2^. The majority of our patients (86%) received GEM‐NabP every two‐weeks. Patients (13.5%; n = 19) that started therapy on a three weeks on one week off regimen were mostly (n = 12) converted to an every two‐week regimen due to toxicity (most frequently neutropenia).

**TABLE 1:** Overall Patient Characteristics

Characteristics	First Line‐GEM‐NabP N (%) N = 140	Second‐Line GEM‐NabP N (%) N = 95	MPACT First‐Line GEM‐NabP N (%) N = 431
Age	Median = 67 yo	Median = 59 yo	Median = 62 yo
	Range = 37‐83 yo	Range = 20‐78 yo	Range = 27‐86 yo
	<65 yo = 40%	<65 yo = 67 (70%)	<65 yo = 254 (59%)
	>65 yo = 60%	>65 yo = 28 (30%)	>65 yo = 177 (41%)
Gender			
Male	82 (58.6%)	50 (52.6%)	245 (57%)
Female	58 (41.4%)	45 (47.4%)	186 (43%)
Pancreatic mass location			
Head	65 (46.4%)	47 (49.5%)	191 (44%)
Body	41 (29.3%)	32 (33.7%)	132 (31%)
Tail	33 (23.6%)	16 (16.8%)	105 (24%)
Undefined	1 (0.7%)		3 (1%)
Ethnicity			
Caucasian	100 (71.4%)	68 (71.6%)	378 (88%)
African American	15 (10.7%)	11(11.6%)	16 (4%)
Hispanic	5 (3.6%)	5 (5.3%)	25 (6%)
Arabic	11 (7.9%)	3 (3.2%)	
Middle Eastern	4 (2.9%)	3 (3.2%)	
Other	5 (3.6%)	2 (2.1%)	12 (3%)
NR		3 (3%)	
ECOG Performance Status			
0	21 (15%)	17 (20.7%)	NR
1	70 (50%)	61 (74.4%)	NR
2	26 (18.6%)	4 (4.9%)	NR
3	1 (0.7%)		NR
NR	22 (15.7%)	13	NR
Karnofsky Performance status score			
100	NR	NR	69 (16%)
90	NR	NR	179 (42%)
80	NR	NR	149 (35%)
70	NR	NR	30 (7%)
60	NR	NR	2 (<1%)
Level of carbohydrate antigen 19‐9^a^			
Normal (< or = 35 U/mL)	24 (17.1%)	14 (14.7%	60 (16%)
<59 × ULN	52 (37.1%)	48 (50.5%)	122 (32%)
>or = 59 × ULN	64 (45.7%)	33 (34.7%)	197 (52%)
Carbohydrate antigen 19‐9 U/ml^a^			
Median	1563	1145	2293.7
Range	<1 −1,381,000	<1 −275,600	1.9‐6,159,233
Metastatic disease sites			
Diffuse	71 (50.7%)	42 (44.2%)	338 (92%)
Single site	69 (49.3%)	53 (55.8%)	33 (8%)
Site of metastatic disease			
Liver	90 (64%)	60 (63.2%)	365 (85%)
Lung	41 (29%)	27 (28.4%)	153 (35%)
Peritoneum	40 (29%)	25 (26%)	60 (14%)

Abbreviations: ECOG, Eastern cooperative oncology group; NR, not reported; yo, years old.

^a^ Carbohydrate antigen 19‐9 level at the start of GEM‐NabP.

**FIGURE 1:**
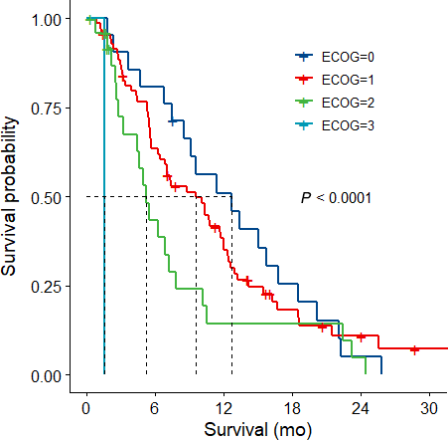
First‐line Gemcitabine plus Nab‐paclitaxel Overall Survival per ECOG performance status

**FIGURE 2:**
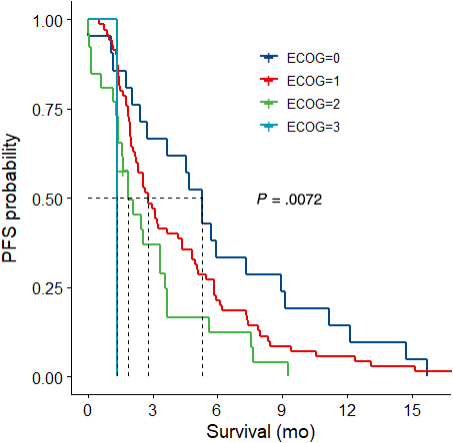
First‐line Gemcitabine plus Nab‐paclitaxel Progression‐Free Survival per ECOG performance status

Outcomes are listed in Table 2. The overall population median OS was 7.5 months. In patients with an ECOG of 0 or 1, median OS was 12.7 months and 9.6 months, respectively (Figure 1). Overall median PFS of the entire population was 2.8 months. In patients with an ECOG of 0 or 1, median PFS was 5.3 months and 2.8 months, respectively (Figure 2). Patients with an ECOG of 2 had a median OS and median PFS of 5.3 months and 1.8 months, respectively (Figure 1 and 2). Disease stability/regression (disease‐control) on first scan was 48.6%. Disease progression was the main reason for patients to stop GEM‐NabP (n = 105; 75%). Other reasons that were termed progression were patient choice/ loss to follow up (n = 8), performance status decline due to cancer or noncancer complication (n = 18), or toxicity (n = 6). Three patients were transitioned to capecitabine maintenance (n = 2) or had yet to progress on GEM‐NabP (n = 1) at the time of data collection. Toxicity reasons for stopping GEM‐NabP were pneumonitis (n = 3), persistent myelosuppression (n = 2), and myalgias (n = 1). No patient deaths were attributed to GEM‐NabP toxicity. For those patients with recorded death dates, the cause of death was attributed to cancer progression due to no further standard treatment options, performance status decline, organ dysfunction not suitable for treatment, complications from cancer (ie, pulmonary embolism, gastrointestinal bleed, hepatic failure, respiratory failure), noncancer related comorbidity (ie, cerebrovascular accident, renal failure), or as a result of the patient's decision to stop therapy.

**TABLE 2:** Efficacy outcomes

Outcome	Result Median (95% CI)
First‐line GEM‐NabP Efficacy	
Overall survival	7.5 mo (6.51‐10.33 mo)
Overall survival ‐ stratified by ECOG performance status	
0	12.7 mo (8.49‐18.49 mo)
1	9.6 mo (6.48‐12.04 mo)
2	5.3 mo (4.41‐10.2 mo)
3	1.6 mo (NA)
	*P* value = <.0001
Progression‐free survival	2.8 mo (2.3‐3.68 mo)
Progression‐free survival stratified by ECOG performance status	
0	5.3 mo (2.73‐9.11 mo)
1	2.8 mo (2.24‐4.34 mo)
2	1.8 mo (1.41‐3.59 mo)
3	1.4 mo (NA)
	*P* value = .0072
Radiographic scan result	
Disease control	68 (48.6%)
Progression/ Toxicity	72 (51.4%)
Second‐Line GEM‐NabP Efficacy	
Overall Survival	7.6 mo (6.12‐8.26 mo)
Overall Survival ‐ stratified by ECOG performance status	
0	8 mo (6.22‐12.99 mo)
1	7.3 mo (5.33‐9.14 mo)
2	6.1 mo (4.61 mo ‐ NA)
	*P* value = .581
Progression‐free survival	2.5 mo (2.14‐3.85 mo)
Progression‐free survival stratified by ECOG performance status	
0	3.5 mo (2.07‐7.24 mo)
1	2.4 mo (2.07‐2.99 mo)
2	2.6 mo (1.74 mo ‐ NA)
	*P* value = .362
Radiographic scan result	
Disease control	38 (40%)
Progression/Toxicity	57 (60%)

Abbreviations: CI, confidence interval; ECOG, Eastern cooperative oncology group; NA, not estimable.

Forty‐six percent (n = 64; 45.7%) received second‐line therapy following progression. Most patients received a fluoropyrimidine‐based combination therapy in the second‐line setting (FOLFIRINOX; 5‐FU plus oxaliplatin; 5‐FU plus irinotecan/liposomal irinotecan). Those with gemcitabine‐based therapy in the second‐line setting generally utilized triplet combination by adding on an additional agent (capecitabine or cisplatin) to GEM‐NabP. Approximately 20% (n = 27; 19.3%) went on to receive third‐line therapy. Third‐line therapy for this group consisted mainly of fluoropyrimidine‐based combination therapy (5‐FU plus oxaliplatin; 5‐FU plus irinotecan/liposomal irinotecan) or a clinical trial.

Only 12.4% of all front‐line patients had a hospital admission while on therapy. Specifically for those who started on the every two‐week regimen (n = 121), 16 patients had admissions. Reasons for admission were mostly related to the disease itself rather than chemotherapy toxicity: gastrointestinal bleed (n = 3); infection without neutropenia (n = 3); failure to thrive (n = 2); pleural effusion (n = 2); thromboembolic event (n = 2), pain (n = 1); congestive heart failure (n = 1), renal failure (n = 1), altered mental status (n = 1). As mentioned above, six patients stopped Gem‐NabP due to toxicity (pneumonitis, persistent myelosuppression, myalgias). GEM‐NabP dose delays or reductions were seen in 24% of patients who started on the every two‐week regimen. Reasons for delays or reduction were due to a single or multiple adverse effect. Only three patients on the every two week regimen had a delay or reduction due to grade 3 hematologic events (grade 3 anemia (n = 2); grade 3 neutropenia (n = 1)). Eighteen patients (12.9%) had growth factor support (pegfilgrastim or filgrastim) added with their treatment to avoid delays in therapy. Common reasons for dose delays or reductions seen with the every two‐week regimen were grade 1 fatigue (n = 2), grade 2 fatigue (n = 6), grade 1 neutropenia (n = 2), grade 2 neuropathy (n = 2), grade 2 myalgias (n = 2), appetite loss (n = 2), and infections (n = 2). All adverse effects requiring dose delays or reductions are listed in Table 3.

**TABLE 3:** Safety concerns

Toxicity	First‐Line GEM‐NabP biweekly regimen only N = 121	Second‐Line GEM‐NabP biweekly regimen only N = 86	MPACT First‐Line GEM/NabP trial N = 421
Number of Admissions	N = 16 (13%)	N = 13 (15%)	NR
	AE leading to death N = 0	AE leading to death N = 0	AE leading to death N = 18 (4%)
Number of Patients with treatment delays	N = 29 (24%)	N = 16 (19%)	41% dose reduction nab‐paclitaxel
			47% dose reduction in gemcitabine
Reasons for dose reductions/delays			
Hematologic			
Grade 1 neutropenia	N = 2 (1.7%)		
Grade 2 neutropenia	N = 1 (0.8%)		
Grade 3 neutropenia	N = 1 (0.8%)	N = 1 (1.2%)	153 (38%)
Hematologic			
Grade 1 thrombocytopenia		N = 2 (2.3%)	
Grade 2 thrombocytopenia	N = 1 (0.8%)	N = 2 (2.3%)	52 (13%)
Grade 3 thrombocytopenia	N = 1 (0.8%)	N = 1 (1.2%)	
Hematologic			
Grade 2 anemia	N = 2 (1.7%)	N = 2 (2.3%)	53 (13%)
Grade 3 anemia			
Nonhematologic			
Grade 1 fatigue	N = 2 (1.7%)	N = 1 (1.2%)	
Grade 2 fatigue	N = 6 (5%)		70 (17%)
Grade 3 fatigue			
Nonhematologic			
Grade 1 liver function test elevation	N = 1 (0.8%)		
Grade 3 liver function test elevation		N = 1 (1.2%)	
Nonhematologic Grade 2 myalgias	N = 2 (1.7%)		
Nonhematologic			
Grade 1 neuropathy			
Grade 2 neuropathy	N = 2 (1.7%)	N = 2 (2.3%)	70 (17%)
Grade 3 neuropathy		N = 3 (3.5%)	24 (6%)
Nonhematologic Grade 3 diarrhea			
Nonhematologic Infections	N = 2 (1.7%)	N = 4 (4.7%)	
Nonhematologic Appetite loss	N = 2 (1.7%)		
Nonhematologic Fever	N = 1 (0.8%)		
Nonhematologic Leukocytosis	N = 1 (0.8%)		
Nonhematologic Failure to thrive	N = 1 (0.8%)		
Nonhematologic Flu like symptoms	N = 1 (0.8%)		
Nonhematologic Dehydration		N = 1 (1.2%)	

### 3.2 Second‐line ‐GEM‐NabP results

Ninety‐five patients received GEM‐NabP in the second‐line setting. The median age was 59 yo. Second‐line therapy population was similar to our first‐line population as a little over half were male (52.6%), most were Caucasian (71.6%), and most had an ECOG performance status of 0‐1 (95%). Patient characteristics and demographics are listed in Table 1. Most common primary tumor location was in the pancreatic head. Forty‐four percent had disease to more than one organ. Most common sites were liver, peritoneum, and lung. Other involved sites were adenopathies, bone, adrenal, ovaries, renal, or rectum. Eighty‐three percent (79 patients) received FOLFIRINOX front‐line. Other front‐line regimens included gemcitabine alone or combined with cisplatin or erlotinib (n = 11), 5‐FU alone or combined with oxaliplatin (n = 4), or one patient that enrolled in a clinical trial. Median dosing for second‐line GEM‐NabP was GEM at 600 mg/m^2^ (range: 400 mg/m^2^ to 750 mg/m^2^) given over 10 mg/m^2^/min +NabP at 125 mg/m^2^ (range: 65 mg/m^2^ to 200 mg/m^2^) over 30 minutes, and 90.5% (n = 86) started this regimen every two‐weeks. Almost all patients (n = 7 out of 9) who started on a three week on one week off schedule were changed to every two‐week after toxicity.

Outcomes are listed in Table 2. The overall population median OS was 7.6 months. In patients with an ECOG of 0 or 1, median OS was 8 months and 7.3 months, respectively. The overall population median PFS was 2.5 months. In patients with an ECOG of 0 or 1, median PFS was 3.5 months and 2.4 months, respectively. Patients with an ECOG of 2 had a median OS of 6.1 months and median PFS of 2.6 months, respectively. Disease stability/regression (disease‐control) on first scan was 40%. The main reason for discontinuation of GemNabP was disease progression (n = 89; 94%). Other reasons were cancer complication (n = 2), patients request (n = 2), and progressive myelosuppression with anemia and thrombocytopenia (n = 2). Reasons for death were similar to the first‐line GemNabP group (cancer progression and no more suitable options or cancer or noncancer related complications). No patient deaths were attributed to GEM‐NabP therapy.

Thirty‐eight percent of patients received subsequent therapy following progression. Third‐line treatment was with fluoropyrimidine‐based combination therapy setting (FOLFIRINOX; 5‐FU plus oxaliplatin; 5‐FU plus irinotecan/liposomal irinotecan), gemcitabine‐based therapy utilizing triplet combination by adding on an additional agent (capecitabine or cisplatin) to GEM‐NabP, or with a clinical trial.

Seventeen percent required admission of all second‐line patients while on therapy. Specifically for those who started on the every two‐week regimen (n = 86), 13 patients had admissions. Main reason of admission was infection (n = 6), followed by failure to thrive/dehydration (n = 2), pain (n = 1), acute renal failure (n = 1), gastrointestinal bleed (n = 1), bowel obstruction (n = 1), and thromboembolic event (n = 1). As seen with first‐line admissions most admissions were related to the patient's disease rather than chemotherapy toxicity. Twenty patients (21.1%) had growth factor support (pegfilgrastim or filgrastim) added with their treatment to avoid delays in therapy. GEM‐NabP dose delays or reductions were seen in 19% of patients who started the every two‐week schedule. Only one patient on the every two‐week regimen had grade 3 hematologic toxicity (grade 3 neutropenia and grade 3 thrombocytopenia). Common reasons for dose delays or reductions (those occurring in more than one patient) seen with the every two‐week regimen were grade 1 or 2 neuropathy (n = 5), infection (n = 4), grade 1 or 2 thrombocytopenia (n = 4), and grade 2 anemia (n = 2). All adverse effects requiring a dose delay or reduction are listed in Table 3.

## 4 DISCUSSION

When looking at our front‐line GEM‐NabP group, our patient demographics were similar to the Von Hoff et al study^8^ (Table 1). Most patients were Caucasian, male, and had a comparable distribution of the primary tumor location. More patients in our population had metastatic disease to only one organ as compared to the Von Hoff trial distribution which commented on metastatic disease site (MPACT = 8%; our population = 49.3%). Although, our descriptions of what qualified as a single site may be the reason for this difference. We had more patients with a poor performance status (MPACT Karnofsky performance status ≤ 70 = < 10%; our population ECOG performance status of 2 was category ~20%) and majority of our patients were ≥65 yo (MPACT ≥ 65 yo = 40%; our population ≥ 65 yo = 60%). Close to 40% of patients in the Von Hoff trial went on to receive subsequent therapy as seen similarly in our study population. Ahn et al performed a recent retrospective review at the Ohio State University of their every two‐week GEM‐NabP front‐line combination (n = 79).^11^ The regimen used was gemcitabine 1000 mg/m^2^ over 30 minutes plus nab‐paclitaxel 125 mg/m^2^ given every two weeks. Demographics were similar in our population to their retrospective review. Outcomes in this analysis showed a median OS and PFS of 10 months and 5.4 months, respectively. Patients in their analysis were excluded if their ECOG PS was ≥2. Kokkali et al performed a similar retrospective observational analysis of their center's GEM‐NabP every two‐week regimen (GEM 1500 mg/m^2^ over 30 minutes + NabP 175 mg/m^2^ given every two‐weeks) patients (n = 46).^12^ Patient characteristics were similar to our population except locally advanced patients and unknown stage patients (17.4% and 26%, respectively) were included in their analysis. Median OS and PFS were 10 months and 5 months, respectively. When we exclude our poor performance status patients (ECOG ≥ 2), our predominantly every two‐week regimen of NabP 125 mg/m^2^ + GEM 600 mg/m^2^ given 10 mg/m^2^/min appeared to maintain a similar efficacy to both of these evaluations.

The starkest difference seen with our regimen in comparison with the above evaluations is in the toxicity profile [8; 11‐12]. Admissions related to toxicity were infrequent in our population along with very few patients experiencing a grade 3 hematologic event (2%) in our study when given this regimen front‐line, whereas in the previous retrospective evaluations discussed by Ahn et al and Kokkali et al grade 3 or higher hematologic toxicity were reported in approximately 30%‐40% of patients. The MPACT trial reported a 38% grade 3 or higher neutropenia, 13% grade 3 or higher thrombocytopenia, and 13% grade 3 or higher anemia (Table 2). Twenty‐four percent of our front‐line patients experienced a toxicity that required either a dose delay or reduction in GEM‐NabP every two‐week, whereas MPACT reported 41% required a NabP dose reduction and 47% required a GEM dose reduction. Toxicity was cut in half likely due to our every two‐week administration and our institution decision to use a lower GEM dose plus a fixed dose rate infusion. Additionally, growth factor support was seen in approximately 13% of our first‐line GEM‐NabP group while MPACT showed 26% had received growth factors. Patients receiving our regimen second‐line after predominantly FOLFIRINOX front‐line showed tolerability and activity.

Our results are not without limitations given the retrospective nature of the evaluation and only results from a single center; however, our results showed a profoundly better hematologic toxicity profile than previously reported results while maintaining efficacy. Our analysis, as far as we are aware, represents the largest population reported retrospectively utilizing an every two‐week GEM‐NabP regimen with the lowest doses proposed. Every two‐week administration allows for the potential of a more patient‐convenient schedule while additionally offering the potential for cost savings as reported by The Ohio State University group which concluded a potential $5,500 a month savings with every two‐week administration.^13^ Patient tolerability and schedule convenience should weigh into treatment decision making for metastatic incurable malignancies, in which therapy is for palliative intent. We suggest the use of an every two‐week GEM at 600 mg/m^2^ administered at a fixed dose rate (10 mg/m^2^/min) plus NabP at 125 mg/m^2^ particularly in patients desiring a modified schedule after a discussion between the provider and patient regarding the modification rationale from Von Hoff's schedule.

## CONFLICT OF INTEREST

Authors do not have relevant conflicts of interest.

## AUTHORS CONTRIBUTION

Jane E. Rogers: Conceptualization, design, formal analysis, data curation, investigation, methodology, resources, supervision, manuscript writing, final approval. Jonathan D. Mizrahi: Conceptualization, design, data curation, investigation, manuscript writing, final approval. Lianchun Xiao: Conceptualization, formal analysis, statistics, manuscript writing, final approval. Chirayu Mohindroo: Data curation, manuscript writing, final approval. Rachna T. Shroff: Conceptualization, design, methodology, resources, provision of study materials or patients, manuscript writing, final approval. Robert Wolff: Resources, provision of study materials or patients, manuscript writing, final approval. Gauri R. Varadhachary: Resources, provision of study materials or patients, manuscript writing, final approval. Milind M. Javle: Resources, provision of study materials or patients, manuscript writing, final approval. Michael Overman: Resources, provision of study materials or patients, manuscript writing, final approval. David R. Fogelman: Resources, provision of study materials or patients, manuscript writing, final approval. Kanwal PS Raghav: Resources, provision of study materials or patients, manuscript writing, final approval. Shubham Pant: Resources, provision of study materials or patients, manuscript writing, final approval. Florencia McAllister: Conceptualization, design, resources, provision of study materials or patients, investigation, methodology, supervision, manuscript writing, final approval.

## Data Availability

Author elects to not share data.
